# Gait analysis using digital biomarkers including smart shoes in lumbar spinal canal stenosis: a scoping review

**DOI:** 10.3389/fmed.2023.1302136

**Published:** 2023-12-14

**Authors:** Tadatsugu Morimoto, Hirohito Hirata, Takaomi Kobayashi, Masatsugu Tsukamoto, Tomohito Yoshihara, Yu Toda, Masaaki Mawatari

**Affiliations:** Department of Orthopaedic Surgery, Faculty of Medicine, Saga University, Saga, Japan

**Keywords:** gait analysis, smart shoes, lumbar spinal canal stenosis, digital biomarker, wearable sensor

## Abstract

Lumbar spinal canal stenosis (LSS) is characterized by gait abnormalities, and objective quantitative gait analysis is useful for diagnosis and treatment. This review aimed to provide a review of objective quantitative gait analysis in LSS and note the current status and potential of smart shoes in diagnosing and treating LSS. The characteristics of gait deterioration in LSS include decreased gait velocity and asymmetry due to neuropathy (muscle weakness and pain) in the lower extremities. Previous laboratory objective and quantitative gait analyses mainly comprised marker-based three-dimensional motion analysis and ground reaction force. However, workforce, time, and costs pose some challenges. Recent developments in wearable sensor technology and markerless motion analysis systems have made gait analysis faster, easier, and less expensive outside the laboratory. Smart shoes can provide more accurate gait information than other wearable sensors. As only a few reports exist on gait disorders in patients with LSS, future studies should focus on the accuracy and cost-effectiveness of gait analysis using smart shoes.

## 1 Introduction

With the advent of an aging society, lumbar spinal canal stenosis (LSS) is a growing and common problem, causing a major health burden worldwide, clinically and socioeconomically (1–8). Although the natural history of LSS is diverse, a progressive loss of function often occurs over time (3, 4). Therefore, early diagnosis and treatment may improve the prognosis of this disease (3, 4).

**Figure 1:**
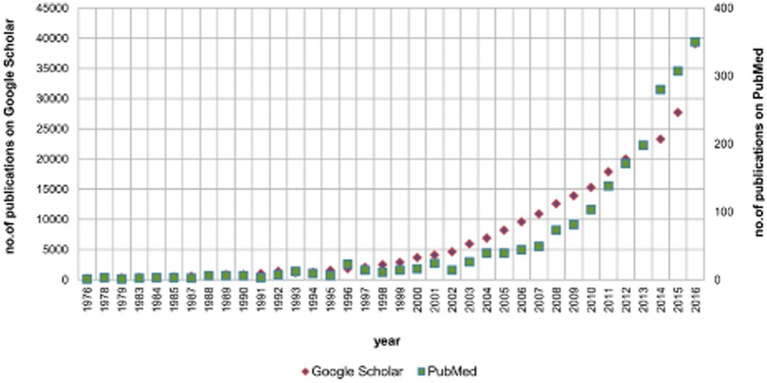
The annual number of publications on gait monitoring with smart shoes using PubMed and Google Scholar. The search criteria included “(gait OR shoe OR walking) AND (inertial OR IMU OR sensor OR wearable).” IMU, inertial measurement unit. Adapted from reference (12) with permission from MDPI.

For early diagnosis of LSS, it is necessary to combine data from various objective biomarkers with self-reported symptoms, standard neurological findings (sensory, motor and reflexes) and imaging studies to improve the accuracy of the diagnostic algorithm. In the further development of digitization throughout healthcare, the more objective term “digital biomarker” has been used to describe this approach in medicine (9–11). Digital biomarkers are classified as physiological indicators (heart rate, pulse, and blood pressure) and behavioral indicators (gait and posture). They are used in fields ranging from sports support to medicine (9–11). Gait is an important biomarker for diagnosing and assessing disease status, as gait patterns are altered in patients with LSS. Objective gait analysis has traditionally been performed in a laboratory, and the recent development and availability of wearable sensor technology have provided a faster, easier, and less expensive method for analysis (3–5). An increasing number of reports have shown that gait analysis using digital biomarkers with wearable sensors can aid in LSS diagnosis, severity, and prognosis (3–5). Wearable sensors, including smartphones, smartwatches, and smart shoes, also known as the Internet of Medical Things (IoMT), are used in medicine and sports owing to their high adherence to daily portable products. Because smart shoes enable a more accurate biomechanical analysis of the ankle joint than smartphones or smart watches owing to the predefined rigid sensor positions in the shoes, studies on gait analysis using smart shoes have increased dramatically in recent years [(12); Figure 1].

However, studies using smart shoes have focused on cardiovascular diseases, sports medicine, and neurological diseases (stroke and Parkinson’s disease), with only a few reports on LSS, although gait abnormalities is a major symptom (4, 5, 13).

This review aimed to provide a scoping review of objective quantitative gait analysis using digital biomarkers in LSS and to note the current status and potential of smart shoes in diagnosing and treating LSS. The scarcity of reports on smart shoes for gait analysis in spinal disease and the heterogeneity of study designs, outcome measures, and variability prevents meta-analyses and adequate systematic reviews. A scoping review cannot locate all relevant literature and cover the scientific literature without bias. Instead, it will discuss the important papers that the authors know about. Thus, this study employed the scoping review method, which allows for a broader, more flexible, and more comprehensive organization and analysis of the existing literature compared to a systematic review.

For this purpose, we also selected many important papers published in peer-reviewed scientific journals and cited extensively the major papers in LSS gait analysis without any deadline restrictions.

## 2 Digital biomarkers in gait analysis

Digital biomarkers that objectively and temporally measure the physiological data of daily life, which were previously difficult to obtain using wearable sensors such as smartphones, smartwatches, and smart shoes, have been attracting attention (9–11). The emergence of digital biomarkers has revolutionized the measurement of physiological data in daily life. Typical digital biomarkers obtained from wearable sensors include vital signs, electrocardiogram, sleep, activity (daily steps, running distance, and calories burned), and gait analysis (9–11). Digital biomarkers obtained from wearable sensors are characterized by their noninvasiveness, long duration (outside the hospital), variety, and large volume of data. Biomarkers are classified according to the timing of the medical intervention: susceptibility/risk biomarkers and diagnostic biomarkers before diagnosis, prognostic/predictive biomarkers and pharmacodynamic/response biomarkers during diagnosis, safety biomarkers during treatment, and endpoint (surrogate) biomarkers and monitoring biomarkers from diagnosis to treatment efficacy (9–11). Therefore, various digital biomarkers derived from gait analysis have the potential to create new clinical value for the diagnosis, treatment, monitoring, and prognostic inference of LSS.

## 3 Trends in gait analysis in the laboratory and beyond

Gait analysis has evolved with technological advances, from purely observational to instrumental methods. Characteristic gait abnormalities observed in LSS include painful claudication and a steppage gait. Observational gait analysis is simple and equipment-free; however, it is inherently subjective, and its validity and reliability depend on the examiner’s skill and experience (14). Objective and quantitative gait analysis helps in understanding the pathophysiology of bipedal walking, identifying treatment focus areas, and optimally monitoring changes in the patient’s condition (15). In the clinical and research fields, the most commonly used simple quantitative assessments are the 10-meter walk test for the most comprehensive index of walking speed, the 6-min walk test for assessing walking endurance, and the Timed Up and Go test for applied walking ability (16, 17). Walking speed affects daily mobility functions directly. Furthermore, walking speed and range of motion of the lower limbs were positively correlated, with 1.0 m/s being the speed at which a person can cross a pedestrian crossing and 0.7 m/s indicating a high risk of falling (15–17). The 6-min walk test and the Time Up and Go test can now be easily measured using free smartphone apps. However, these simple assessments do not specifically identify the aspects of gait that differ from those of a healthy gait.

In contrast to performance measures such as gait speed, instrumental quantitative gait analysis contributes to identifying causes that impair bipedal stability and efficiency and events and conditions that should be focused on during treatment. Instrumental quantitative gait analysis is commonly performed according to standard methods based on kinematic analysis of the displacement of body parts during walking (three-dimensional (3D) motion analysis), kinematic analysis of the external forces acting on the body (ground reaction forces), and electromyographic analysis of the muscle activity involved in the walking movement to examine gait parameters, such as spatial (length), temporal (duration), or derived indices (asymmetry, variability) (3, 15). Because these measures can be obtained using multiple inputs from different gait sites, they show high recognition rates and are crucial for classifying and quantifying gait disorders (3, 15, 16). Kinematic measurements can be obtained from any recording device linked to a computer (e.g., motion capture systems or inertial measurement units). The 3D analysis focuses on body movements, and the mainstream approach is optical. Markers attached to various body parts are photographed using a semiconductor camera, and the displacement, angular velocity, angular acceleration, stride length, and stride width of joint movements are calculated (18). Commonly used spatiotemporal gait metrics for quantitative evaluation include spatial (step and stride length) and temporal (step and stride time) parameters, spatiotemporal (walking speed and cadence: composite parameters derived from spatial and temporal variables) parameters, gait asymmetry, gait variability (Table 1), and joint angles (3).

**Table 1:** Spatiotemporal gait metrics: spatial, temporal, spatiotemporal, gait asymmetry, gait variability.

Type	Parameters	Definition	Units
Spatial	Step length	Average distance between two consecutive contacts of any foot with the ground	Meters (m)
Spatial	Stride length	Average distance between two consecutive contacts of the same foot with the ground	Meters (m)
Temporal	Step time	Average time between two consecutive contacts of any foot with the ground	Seconds (s)
Temporal	Stride time	Average time between two consecutive contacts of the same foot with the ground	Seconds (s)
Spatiotemporal	Walking speed (or gait velocity)	Average distance traveled per second	Meters/second (m/s)
Spatiotemporal	Cadence	Average rate (or frequency) of steps	Steps/minute
Gait asymmetry	Step time asymmetry	Average difference in time taken for successive steps on the left and right foot	Seconds (s)
Gait asymmetry	Step length asymmetry	Average difference in length for successive steps on the left and right foot	Meters (m)
Gait variability	Step time variability	Step-to-step variability of step time	Standard deviation (SD) coefficient of variance (cov = SD/mean)
Gait variability	Step length variability	Step-to-step variability of step length	Standard deviation (SD) coefficient of variance (cov = SD/mean)
Gait variability	Walking speed (or gait velocity) variability	Step-to-step variability of walking speed	Coefficient of variance (cov = SD/mean)

**Figure 2:**
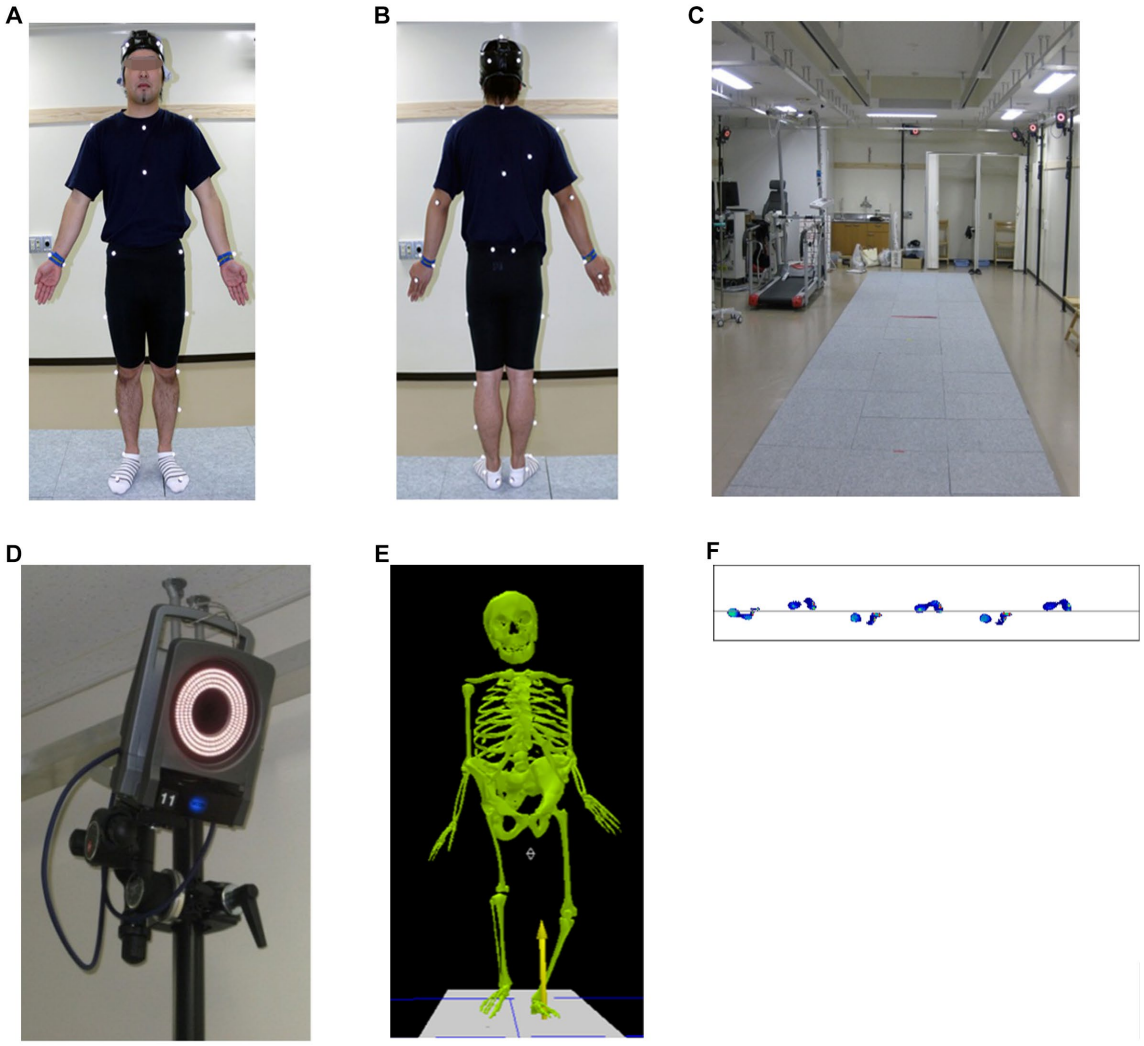
Vicon Motion System™, Oxford, UK. **(A,B)** Thirty-five infrared reflective markers are attached to the body surface. **(C)** Patients were asked to walk freely on an approximately 8 m walking path with a ground reaction force meter installed in the center of the path **(D)** and photographed by 14 infrared cameras. The infrared reflective markers were positioned using the plug-in-gait model at Saga University. The video motion and ground (foot) force reaction data were seamlessly merged to enable spatiotemporal and dynamic evaluation of gait abnormalities **(E,F)**.

For kinetic analysis, ground (foot) force reaction (GRF) analysis, including foot pressure analysis, was used to measure the magnitude, direction, and location of the application (19, 20). Adding 3D analysis data to GRF or electromyography data can provide a more comprehensive depiction of the gait. The marker-based system device is the traditionally used and highly accurate method, which combines 3D motion analysis (video analysis, optical motion tracking and analysis, multi-sensor, or gyroscope), electromyography, and GRF analysis in the laboratory for gait analysis (i.e., VICON) [Figure 2; (19, 20)].

A combined analysis of 3D motion and digital biomarker data obtained from ground reaction forces and electromyograms will improve understanding of the indices of spatial and temporal factors in the gait cycle, characteristics of the center of gravity movement that contribute to gait efficiency, and the relationship between joint motion and muscle activity in the lower limbs and trunk. However, laboratory gait analyses, including marker-based 3D motion capture systems, GRF, and electromyography, have disadvantages regarding space, equipment, time, workforce, cost, technical expertise, and exhaustive data analysis, making their clinical application difficult (21). There is also the problem of the “Hawthorne effect” in which people consciously alter their gait because they know that they were monitored (21) and the “white coat effect” (22), in which tension in an unfamiliar environment can alter patient performance. In addition, marker-based gait analysis requires subjects to expose their skin for accurate marker placement to obtain more accurate data, which may cause inconvenience (23). Recently, the accuracy of markerless 3D measurements, such as Media Pipe^1^ and OpenPose,^2^ has improved; these require no expertise or special cameras, are free for noncommercial use, and are expected to expand opportunities for clinical applications (23). Notably, lower limb range of motion (ROM) was measured in the sagittal plane using OpenPose from images taken with a single digital camera (23). Although OpenPose cannot substitute a complete 3D motion analysis system, it can be used for gait analysis (23). OpenPose is a markerless system without special cameras, thus reducing analysis costs and time. Thus, the development and increased availability of wearable sensor and video analysis technology, especially markerless systems using human posture tracking algorithms, has provided a faster, easier, less expensive, and more representative way to measure regular walking patterns (or ‘free-living’ gait) outside the laboratory as an alternative to marker-based gait analysis in the laboratory (3–5, 24).

Wearable sensors and markerless 3D measurement can provide a more accurate assessment of a patient’s gait and posture in “everyday life,” which may not be reflected in tests performed by a physician in the hospital or outside the laboratory. Therefore, combining wearable sensors and markerless 3D measurement (OpenPose, Media Pipe) could be a “game changer” in motion and gait analysis.

## 4 Summary of publications on objective quantitative gait analysis using digital biomarkers in LSS

The most characteristic clinical presentation of LSS is neurogenic intermittent claudication, which causes pain and numbness from the buttocks to the lower extremities on one or both sides during walking, resulting in a slower walking speed and shorter total walking distance (3, 25). A systematic literature review by Wang et al. in 2022 revealed that most conventional quantitative gait analyses of LSS were performance-oriented studies on walking speed and distance, such as motorized treadmill trials (24 publications) and timed up-and-go trials (19 publications) (24).

Patients with LSS often have postures that cause the lumbar spine to flex more to maximize spinal canal volume and minimize pain and symptoms during walking, leading to postural abnormalities (25, 26). In addition to lower-extremity pain, muscle weakness and sensory disturbances can result in balance dysfunction (24, 26, 27). Furthermore, changes in sagittal spinal alignment may affect the hips (28, 29) and knees (30). Kinematic (3D motion analysis), kinetic (GRF), and electromyographic (EMG) analyses of gait can produce abnormalities in spatial, temporal, or derived indices (asymmetry and variability) of gait. These observations were made upon reflecting on these LSS-induced lower-extremity neuropathies and alignment abnormalities in the spine and lower-extremity joints from objective quantitative gait analysis using instruments (3, 16).

Table 2 summarizes the publications on objective quantitative gait analysis using digital biomarkers in LSS. Although most studies have investigated spatiotemporal gait metrics (spatial, temporal, spatiotemporal, gait asymmetry, gait variability), only a few investigated trunk and lower-extremity joint angles, plantar pressure distribution, and EMG (Table 3).

**Table 2:** Summary of publications on objective quantitative gait analysis using digital biomarkers in LSS.

Reference	Year	Nationality	Product	Instrumentation	Wearable sensor location	Environment
(31)	2022	China	Footscan® pressure plate 13 (RSscan International, Olen, Belgium)	GRF plate		Indoor 10 m circular track
(32)	2022	Czech Republic	11 infrared cameras Oqus 300 and 300+, two force platforms (Kistler type 9281EA, Kistler Group, Winterthur, Switzerland)	Motion capture, GRF plate		Laboratory
(22)	2021	Australia	MetaMotion C (MbientLab Inc., CA, USA)	Motion capture, accelerometer, gyroscope, magnetometer	Sternal	Indoor hospital ward
(33)	2021	China	IDEEA (MiniSun, LLC, Fresno, CA, USA)	Accelerometer (acceleration electronic sensors)	fourth metatarsal, thigh, sternal	Indoor horizontal walkway
(34)	2020	China	Footscan® 3D pressure system (RSscan International, Olen, Belgium)	GRF plate		Indoor 10 m circular track
(35)	2020	USA	Shimmer3 wearable sensor platform (Shimmer Sensing, Dublin, Ireland)	Accelerometer, gyroscope, magnetometer		NA
(36)	2020	Switzerland	RehaGait® system (Hasomed GmbH, Magdeburg, Germany)	Accelerometer		Indoor hospital ward
(37)	2020	Korea	Human Track®, Gait & Motion Analysis System (RBiotech Co., Ltd., Seoul, Korea), FreeStep software® (Sensor Medica, Rome, Italy)	Accelerometer, gyroscope, magnetometer		Laboratory
(21)	2020	Australia	NA	Videography		NA
(38)	2018	Switzerland	RehaGait® system (Hasomed GmbH, Magdeburg, Germany)	Accelerometer, gyroscope, magnetometer	Lateral shoe, lower and upper legs, pelvis	Indoors (clinic)
(39)	2018	Switzerland	RehaGait® system (Hasomed GmbH, Magdeburg, Germany)	Accelerometer, gyroscope, magnetometer	Lateral shoe, lower and upper legs, pelvis	NA
(40)	2018	China	IDEEA3; MiniSun (LLC, Fresno, CA, USA), GoPro Hero3 high-speed camera (GoPro, San Mateo, CA, USA)	Accelerometer, gyroscope, magnetometer	Chest, thigh, ankles, and plantar surface of foot	Indoor hospital ward
(4)	2017	USA	Smart shoes (UCLA Wireless Health Institute) with pressure sensors (FSR400, Interlink Electronics, USA)	GRF (smart shoes)	Shoe (heel, lateral plantar, toe)	Laboratory
(41)	2017	Japan	Vicon MX system® (Vicon Motion Systems, Oxford, United Kingdom) 8cameras, round force platform (AMTI, model OR-06; Advanced Mechanical Technology, Watertown, MA, USA); Telemyo 2,400 T (Noraxon, Scottsdale, AZ, USA)	Motion capture, GRF plate		Laboratory
(42)	2015	Japan	NA	Videography		Laboratory
(43)	2014	Brazil	MX40 Vicon system (Vicon, Oxford, UK)	Motion capture		Indoor horizontal walkway
(44)	2014	Japan	Triaxial accelerometer (WAA-066, ATR Promotions Co., Japan)	Accelerometer	Lumbar and cervical spines	Laboratory
(45)	2013	USA	Long instrumented walkway (GaitRite®; CIR Systems, Inc., Havertown, PA, USA); electromagnetic tracking system (Liberty, Polhemus Inc., Colchester, VT, USA).	GRF plate		Laboratory
(46)	2002	Japan	NA	GRF plate		Indoor 10 m circular track

NA, not applicable; m/w, men/ women; yrs, years old; GRF, Ground reaction force; SGM, Spatiotemporal gait metrics (spatial, temporal, spatiotemporal, gait asymmetry, gait variability); Kinematic variable, Trunk or Lower joint angle and range of motion; Kinetic variable, Vertical force, pressure distribution, and center of force on foot; EMG, Electromyography.

**Table 3:** Summary of publications on spatiotemporal gait metrics, Kinematic and Kinetic variable and EMG in LSS.

Reference	Patient characteristics (N), gender (m/w), Mean age (yrs), study	Variable	Study findings
(31)	*N* = 31 (NA), 60 yrs, LSS patients vs. controls	SGM	↑The medial-lateral center of pressure with increasing distance
(32)	*N* = 15 (11/4), 62 yrs, LSS patients vs. controls	SGM Kinematic variable, Kinetic variable	↓stride length, step length, step times, cadence, swing times ↑stride width, stance times, initial double limb support
(22)	*N* = 25 (17/8), 59 yrs, LSS patients vs. controls	SGM	↑ step length and step time asymmetry ↓stride time, step time, and cadence, stride length and step length
(33)	*N* = 49 (18/31), 80 yrs, LSS patients vs. controls	SGM Kinetic variable	↑small intermittent claudication, single support, double support, step duration, and pulling accel ↓Push off, speed, step length, and Stride length
(34)	*N* = 20 (12/8), 60 yrs, LSS patients vs. controls	Kinematic variable, Kinetic variable	↑foot contact time for LSS,↑foot progression angle for LSS, ↑pressure time integral in forefoot, medial and lateral heal for LSS
(35)	*N* = 10 (3/7), 70 yrs, LSS patients vs. controls, Knee osteoarthritis vs. controls	SGM	Foot flat ratio, gait speed, stride length and cadence were identified as the best gait characteristics for the LSS population discrimination. Normal paced walking tests (6MWT, SPWT) are better suited for distinguishing gait characteristics
(36)	*N* = 29 (17/12), 73 yrs, LSS patients vs. controls	Kinematic variable	↑ vertical pelvis acceleration for pre-op, 10wks, and 1 yr. ↓ AP and ML pelvis acceleration for pre-op, 10wks, and 1 yr
(37)	*N* = 17 (3/14), 66 yrs, LSS patients vs. controls	Kinematic variable, EMG	↑peak knee varus angle for LSS ↑tensor fascia and↓ quadriceps muscle activity for LSS: LSS patients required increased activation of hip abductors and recruited neighboring quadriceps muscle fibers when performing hip abduction.
(21)	*N* = 15 (8/7),73 yrs, LSS patients vs. controls	SGM	↓cadence, step length, gait velocity, ↑step time(a decrease in gait speed and cadence is caused by the presence of lower limb pain and dysesthesias)
(38)	*N* = 29 (17/12), 73 yrs, LSS patients vs. controls	SGM	↓gait velocity, gait length, ↑gait duration and gait asymmetry
(39)	*N* = 19 (11/8), 74 yrs, LSS patients vs. controls	SGM	↑change in acceleration pattern for 1 yr. ↑ change in acceleration variability for pre-op, 10wks, 1 yr. ↑ change in acceleration pattern and quality for pre-op, 10wks, 1 yr
(40)	*N* = 20 (NA), 58 yrs, LSS patients vs. controls	SGM	↓step length and stride length
(5)	*N* = 15 (4/11), 58 yrs, LSS patients	SGM, Plantar pressure distribution	
(20)	*N* = 6 (5/1), 69 yrs, LSS patients (pre-, post operation)	SGM, Plantar pressure distribution, EMG	↓ (Kinematic analyses) thorax angle, pelvic angle(tendency, not significant), (EMG)the activity of the PVM ↑ (Kinematic analyses) Cadence, gait velocity, knee flexion angle,(Kinetic analyses),Hip and Knee flexion torques, (EMG)The activity of the VL
(42)	*N* = 7 (5/2), 71 yrs, LSS patients vs. Hip oseoarthritis	SGM	↑sagittal plane knee ROM during stance
(43)	*N* = 14 (10/4),75 yrs, LSS patients vs. controls	SGM	↓stride length and gait velocity ↑anterior trunk tilt
(44)	*N* = 11 (8/2), 73 yrs, LSS patients	SGM	↑postural sway
(45)	*N* = 25 (11/14), 73 yrs, LSS patients	SGM	↓gait velocity
(46)	*N* = 60 (11/29), 63 yrs, LSS patients (cauda equina and radicular type)	SGM	Abnormalities of various factors related to the style of walking soon after the patients began to walk

NA, not applicable; SGM, Spatiotemporal gait metrics (spatial, temporal, spatiotemporal, gait asymmetry, gait variability); Kinematic variable, Trunk or Joint angle and range of motion; Kinetic variable, Vertical force, pressure distribution, and center of force on foot; EMG, Electromyography.

The characteristics of gait deterioration in patients with LSS compared to those in healthy subjects include decreased gait velocity (35, 38, 42, 44, 46), decreased time or length of gait (step or stride) (21, 22, 28, 32, 33, 35, 38, 40, 44), decreased cadence (21, 22, 35, 42), gait asymmetry (38), and prolonged gait duration (21, 22, 32, 35, 38). Kinematic analysis showed that LSS decreased hip ROM (42), increased knee ROM (42) and lumbar flexion (anterior trunk tilt) in the sagittal plane (44), and increased the foot contact time and progression angle (34). This observation may be due to neuropathy (muscle weakness and pain) in the lower extremities caused by LSS. For the EMG variables, muscle activity in the LSS was higher in the tensor fascia, quadriceps (37), and vastus lateralis muscles (20). Additionally, muscle activity was lower in the paravertebral muscles (20) of patients with LSS than in healthy controls (Table 4). Although the number of reports on the gait analysis of LSS using wearable sensors has increased (33, 44), only two studies on smart shoes were written by the same authors (4, 5).

**Table 4:** Characteristic of gait analysis on patients with lumbar spinal canal stenosis.

Gait type	Neurogenic intermittent claudication, painful limp, steppage gait
Spatiotemporal gait metrics	Decreased gait velocity (21, 35, 38, 42, 44, 46), decreased time or length of gait (step or stride) (21, 22, 32, 33, 35, 38, 40, 44), and decreased cadence (42), prolonged gait duration
Kinematic variable	Decreased hip and knee range of motion (42) Lumbar flexion (anterior trunk tilt) in the sagittal plane (20, 43, 44)
Kinetic variable	Increased knee flexion torques (20)
Electromyography	Muscle activity in the LSS was higher in the tensor fascia, quadriceps (37), and vastus lateralis muscles (20) and lower in the paravertebral muscles (20).

## 5 Smart shoes: status quo and quo vadis

Smart shoes are ordinary shoes with technological innovations, such as biometric data recording and automatic size adjustment according to the individual (13). Shoes with at least one actuator or sensor built in are “smart.” Leading companies have developed smart shoes incorporating various technologies, including pressure sensors, accelerometers, gyro sensors, piezoelectric pedometers, and Bluetooth. These smart shoes can analyze posture, gait patterns, and ankle momentum and measure the number of steps and calories burned via a smart app (13); they include Lechal Shoes that navigate using GPS (13, 47), Google’s talking shoes (48), Adidas’ Micropacer (49), Nike’s Adapt BB, Puma’s Fit Intelligence, Samsung’s IOFIT, and Asics’ EVORIDE ORPHE.

**Figure 3:**
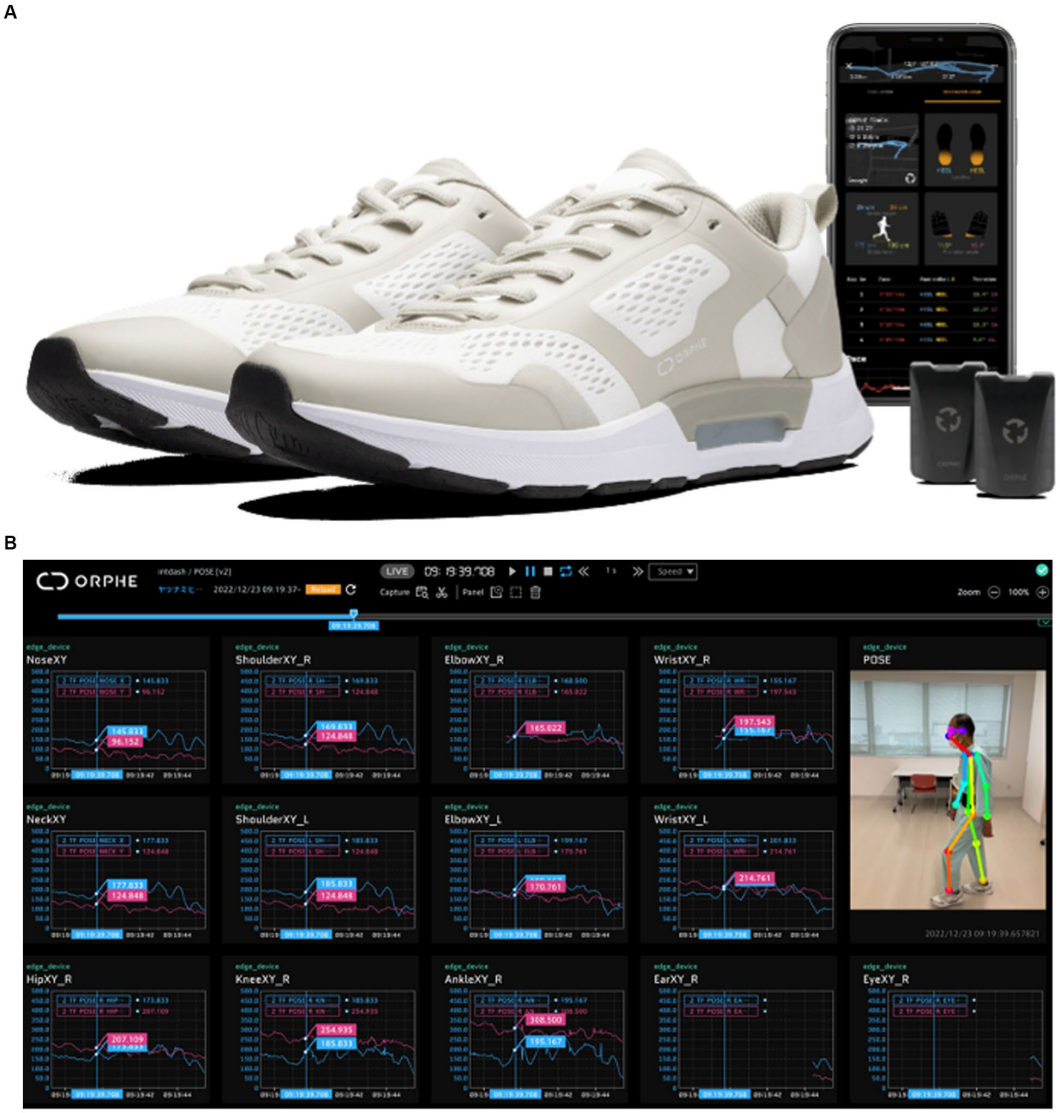
Asics’ EVORIDE ORPHE smart shoes can measure the time of each segment of the gait cycle, landing and departure angles, spatiotemporal evaluation of gait using 6-axis (3-axis acceleration, 3-axis angular velocity) motion sensors built into the plantar surface, and indicators for gait evaluation such as ankle joint angle and plantar pressure [landing impact, ground (foot) force reaction]. **(A)** Is adapted from https://orphe.io/presswith permission of ORPHE. **(B)** Linkage with 3D motion analysis was done by linking the videos captured by a single digital camera with multifaceted gait analysis using OpenPose.

The shoe incorporates pressure, acceleration, and gyroscope sensors to track the user’s activity. Real-time feedback can be provided by connecting it to a personal computer or smartphone. Asics’ EVORIDE ORPHE enables multifaceted gait analysis by linking 3D motion analysis using OpenPose from videos captured by a single digital camera with kinematics and GRF data obtained from smart shoes (Figure 3). However, no comparisons have been made between marker-based 3D movement analysis (numerous video cameras and infrared markers) combined with GRF measurements in the laboratory (Figure 2) and markerless 3D movement analysis outside the laboratory using low-cost and convenient smart shoes and a single digital camera on a smartphone in patients with LSS. This aspect requires further exploration.

Biofeedback systems combined with smart shoes can prevent injuries in runners (50, 51), prevent and detect falls in older patients (50, 52), monitor posture in patients with back pain (52), and detect gait abnormalities in osteoarthritis to prevent joint damage (53). Moreover, Bluetooth-and Wi-Fi-capable smart shoes can help the visually impaired navigate their destinations using Google Maps functionality (13, 54). Smart shoes are a useful tool for evaluating gait analysis because they (1) have predefined rigid sensor positions on the soles for accurate and flexible biomechanical analysis, (2) can monitor the highly fixed movement of gait and automatically assess functional biomechanics, and (3) are discreet and non-stigmatizing to incorporate, improve patient acceptance and long-term adherence, and allow gait to be assessed spatiotemporally and mechanically (12). When comparing the accuracy of the number of steps by wearing the sites at the hip, buttock, thigh, ankle, and wrist, the ankle joint showed the highest accuracy (55). Therefore, smart shoes are more suitable as wearable sensors for gait analysis than smartphones or smartwatches because they provide more gait information (gait asymmetry and GRF) (4, 5, 12, 56).

Studies on smart shoe gait analyses have increased dramatically in recent years (12). However, they have focused on cardiovascular diseases, sports medicine, and neurological diseases (stroke and Parkinson’s disease), with only a few on degenerative spinal diseases, although gait abnormalities is a major symptom (4, 5, 12). This may be because wearables have only recently emerged as practical tools to assist health management. Smart shoes enable the long-term recording and analysis of superficial information, including walking distance, walking time, and calories burned, which can be obtained from smartphones and smartwatches, and stride length, landing angle and impact, the area where the foot touches the ground, and changes in walking style (4, 5, 12, 56). Smartphones may motivate runners and patients to exercise by encouraging behavioral changes through daily step challenges and goal setting. Furthermore, insole-based systems can easily measure several parameters related to lower-extremity health, such as plantar pressure, body temperature, pulse rate, and gait dynamics (4, 5, 12). Thus, these data-collecting smart shoes are similar to the IoMT.

Accumulating gait data and machine learning algorithms may help establish a warning system for faster and better fall response. Therefore, accurate gait analysis data from smart shoes can help in the early detection, assessment of fall risk, treatment decisions, monitoring of treatment, and outcome evaluation of diseases that cause gait disorders, including LSS. Outcome measurements will shift from being subjective to combining subjective and objective measurement tools derived from digital biomarkers. Information from wearable sensors other than smart shoes will be integrated with artificial intelligence to provide useful information for healthcare providers regarding treatment. With the entry of major shoe companies, market penetration of smart shoes with high comfort and convenience is expected to increase rapidly. However, reports on the efficacy of smart shoes for gait analysis in LSS, usability, data security, and cost-effectiveness are lacking (57). The legal system may be unable to keep pace with advances in connected medical product technology, and data security must be a top priority, particularly concerning patient information.

## 6 Conclusion

Proper diagnosis and treatment of LSS require objective and subjective methods of assessment. Objective quantitative gait analysis and subjective patient assessment are useful for diagnosis, prevention, therapeutic intervention, treatment management, and outcome assessment. Although objective quantitative methods of gait analysis have been performed using laboratory-based 3D motion analysis, ground reaction force, and electromyography, challenges may occur regarding workforce, time, expertise, and cost. Wearable sensor technology (especially smart shoes) and markerless motion analysis systems have made it possible to replace conventional gait analysis with markers in the laboratory, which is faster, simpler, cheaper, and more reflective of everyday life. Using smart shoes for gait analysis shows great potential; however, evaluating their accuracy and cost-effectiveness is crucial. Future studies should aim to address these concerns and provide more insight into the use of smart shoes for gait analysis in the diagnosis, treatment management, and outcome assessment of LSS. These advances in technology and methods will help healthcare professionals provide better care for patients with LSS.

## Author contributions

TM: Conceptualization, Data curation, Investigation, Writing – original draft. HH: Conceptualization, Methodology, Supervision, Visualization, Writing – original draft. TK: Data curation, Formal analysis, Writing – original draft. MT: Supervision, Validation, Visualization, Writing – original draft. TY: Supervision, Validation, Writing – original draft. YT: Investigation, Methodology, Writing – original draft. MM: Supervision, Validation, Writing – review & editing.

## Footnotes

^1^ https://google.github.io/mediapipe/

^2^ https://cmu-perceptual-computing-lab.github.io/openpose/web/html/doc/index.html
